# Involvement of circRNAs in the Development of Heart Failure

**DOI:** 10.3390/ijms232214129

**Published:** 2022-11-16

**Authors:** Grażyna Sygitowicz, Dariusz Sitkiewicz

**Affiliations:** Department of Clinical Chemistry and Laboratory Diagnostics, Medical University of Warsaw, 02-097 Warsaw, Poland

**Keywords:** circRNA, heart failure, miRNA sponge, cardiac hypertrophy, cardiac fibrosis

## Abstract

In recent years, interest in non-coding RNAs as important physiological regulators has grown significantly. Their participation in the pathophysiology of cardiovascular diseases is extremely important. Circular RNA (circRNA) has been shown to be important in the development of heart failure. CircRNA is a closed circular structure of non-coding RNA fragments. They are formed in the nucleus, from where they are transported to the cytoplasm in a still unclear mechanism. They are mainly located in the cytoplasm or contained in exosomes. CircRNA expression varies according to the type of tissue. In the brain, almost 12% of genes produce circRNA, while in the heart it is only 9%. Recent studies indicate a key role of circRNA in cardiomyocyte hypertrophy, fibrosis, autophagy and apoptosis. CircRNAs act mainly by interacting with miRNAs through a “sponge effect” mechanism. The involvement of circRNA in the development of heart failure leads to the suggestion that they may be promising biomarkers and useful targets in the treatment of cardiovascular diseases. In this review, we will provide a brief introduction to circRNA and up-to-date understanding of their role in the mechanisms leading to the development of heart failure.

## 1. Introduction

The studies in the last decade have demonstrated that 80% of our genome is transcribed, but only 2% of the RNA constitutes protein-coding molecules [1]. This crucial discovery made 10 years ago suggested that a definite majority of our genes are transcribed to protein non-coding RNA (ncRNA). Many ncRNA categories have been characterised and classified. The most frequently used classification takes into account the size of molecules. Shorter (<200 nucleotides) are classified as small ncRNAs, including microRNA (miRNA), while those exceeding 200 nucleotides are regarded as long non-coding RNAs (lncRNAs). The lncRNA family includes both linear and circular forms of RNA. Although it was earlier believed that circular RNAs (circRNAs) occur rarely compared with linear information RNAs, the recent studies have demonstrated that circRNAs can be relatively abundant and are the predominant transcripts of even hundreds of genes [2]. These circular molecules are ubiquitous in humans and until now more than 30,000 different circRNAs have been identified [3]. The fact is however alarming that they contribute to the development and progression of many diseases, playing an important role in the pathogenesis of cardiovascular diseases.

Cardiovascular diseases (CVDs) are still the main cause of death and invalidity worldwide [4]. In spite of vast progress in the treatment of CVDs, a full use of RNAs in the therapy and diagnostic procedures is still not possible in view of the encountered technical difficulties, which decelerate and/or even render their full clinical use impossible. Recent studies have suggested a potential of miRNA molecules in the treatment of patients with CVDs [5,6,7]. LncRNAs have also emerged as potential diagnostic and therapeutic targets in CVDs [8,9]. The information of the role of circRNAs in the development of heart diseases and their importance as the diagnostic biomarkers and also new therapeutic trends is still insufficient.

## 2. Biogenesis and Functions of circRNAs

**Figure 1:**
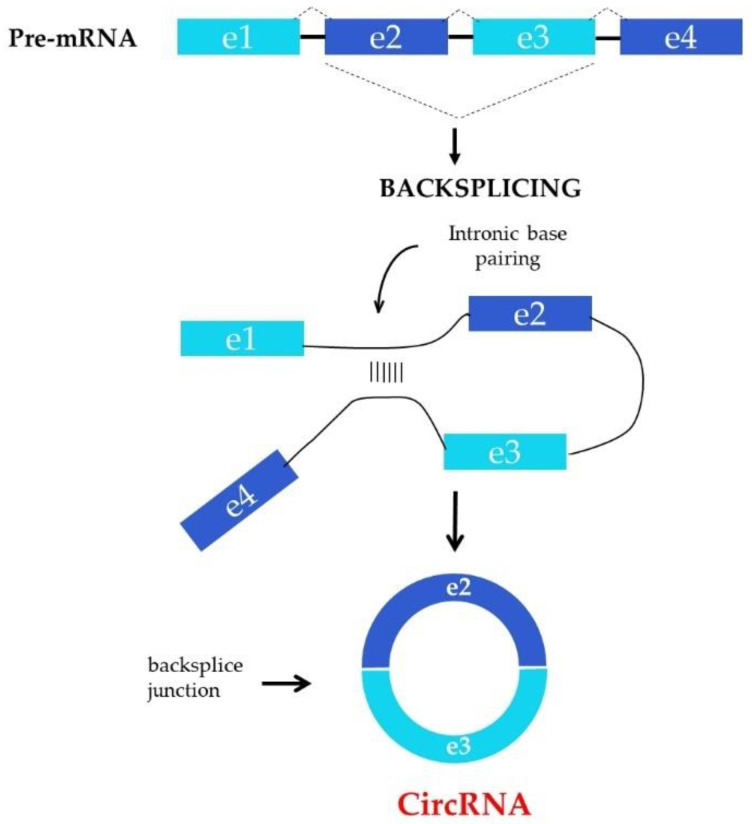
Biogenesis of circRNA. The spliceosome mechanism that normally catalyzes the linear splicing of pre-mRNA s can also perform a back-splicing reaction between two exons, resulting in the formation of circRNA. Back-splicing uses the same canonical splicing machines and canonical splicing sites that are needed for linear splicing. Mechanically, backsplicing requires that the donor and acceptor sites of backspliced exons are in close proximity to each other. This can be achieved by direct pairing of RNA bases of inversely complementary sequences in the introns flanking the backspliced exons which bind to these flanking introns.

The precise mechanisms underlying the biogenesis of circRNAs still remain unclear. Several possible biogenesis mechanisms have been proposed as yet. The spliceosomal machinery is regarded as the basic procedure of circRNAs formation. Reversed, repeating elements and RNA-binding proteins can connect donor-splice and acceptor-splice sites through appropriate pairing of bases [10] or binding to definite motifs [11], what contributes to intron looping. Then, exonic circRNA (ecircRNA) or exon-intronic circRNA (eicircRNA) are formed after intron splicing. The next significant mechanism of circRNAs biogenesis is lariat formation. The spliced lariat after formation of terminal mRNA can contain exons due to alternative splicing (omitting of exons) or only introns, which leads to biogenesis of ecircRNA or circular intronic RNA (icircRNA) [12]. Simplified spliceosome machinery is present in Figure 1.

**Figure 2:**
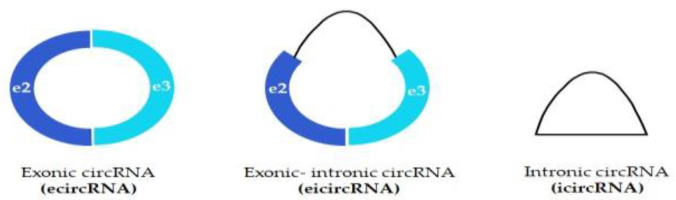
Different types of circular RNAs.

In this respect, circRNAs can be divided into three types depending on the derivative of components: ecircRNAs (formed from exons) [13], icircRNAs (formed from introns) [14] and eicircRNAs (formed both from exons and introns) [15] (Figure 2). Most of the known circRNAs are formed from exons, while ecircRNAs and eicircRNAs only constitute their small pool [16].

**Figure 3:**
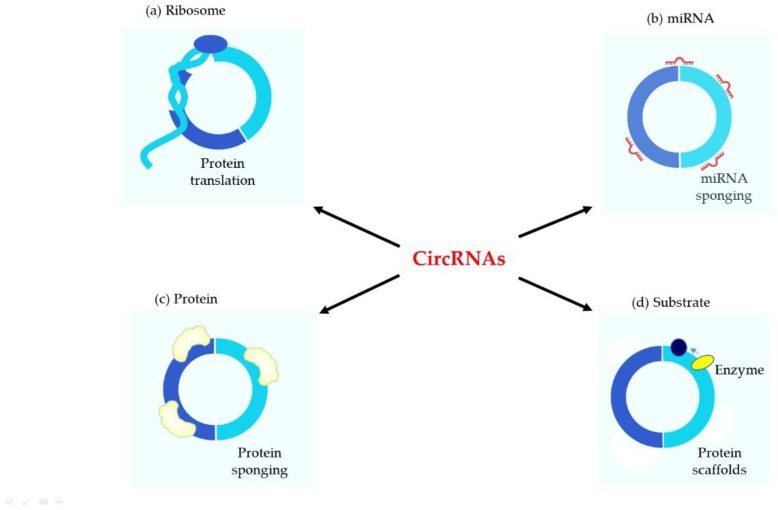
Functions of circular RNAs. (**a**) Ribosome. In the presence of internal ribosomal enter sites and a corresponding open reading frame, circRNAs can affect protein translation. (**b**) miRNA. CircRNAs can sponge the miRNAs to act as a cytoplasmic miRNA inventory. The scavenging of miRNAs removes the repression of target RNAs leading to an increase in their translation (in the case of mRNA) or activity (in case of lncRNA). (**c**) Protein. CircRNAs can act as a sponge for cytoplasmic proteins, retain certain transcription factors in the cytoplasm, or serve as a carrier for the transport of these molecules. (**d**) Substrate. CircRNAs can act as a scaffold for enzymes leading them to specific locations.

Compared with miRNA and lncRNA, studies on circRNA are still insufficient and require much commitment. It has been demonstrated that circRNAs play an important role at many developmental stages and pathophysiological conditions: acting as miRNA sponges [17], interacting with RNA-binding proteins (RBP) [18], acting as transcription or translation regulators [15], affecting pre-mRNA splicing [19] and also participating in translation of proteins [20] (Figure 3). The most frequently described function of circRNAs is the activity as a miRNA sponge. The term “miRNA sponges” highlights the function of circRNAs as competitive inhibitors, which contain multiple miRNA-binding sites and prevent miRNAs from binding to mRNA targets. CircRNAs comprise miRNA response elements (MREs), which advance binding between circRNA and miRNA. Such binding leads to a reduction of the target gene expression through degradation of RNA-induced silencing complex, or through limitation of mRNA translation [21,22,23]. This reaction can also reduce the level of functional miRNAs and increase the expression of target mRNAs [21,24].

The term “miRNA sponges” stresses the function of circRNAs as competitive inhibitors, which contain many miRNA-binding sites and prevent miRNA binding to the target mRNA. A significant breakthrough was the study by Memczak et al. [25], in which CDR1as and its binding sites for miR-7 were described. Since that time the function of circRNAs as miRNA sponges has attracted the interest of many biologists, calling the attention to the extensive influence of circRNAs on the activity of various miRNAs. Here, are some of the more important examples. It has been demonstrated that circFOXk2 promotes cell growth, migration, invasion and apoptosis—binding to many sites and acting as a miR-942 sponge [26]. CircALMS1_6 can participate in the regulation of heart remodelling, functioning as a miR-133 sponge [27].

Apart from miRNA sponges, circRNAs also can function as protein sponges (Figure 2), i.a. as RBP sponges, and RBPs can also participate in back-splicing [28,29,30,31,32,33]. RBPs, being the proteins participating in the transcription and translation of genes, enter interactions with circRNAs, leading to inhibition of RBP proteins [33,34]. CircMbl absorb MBL proteins and regulate further physiological processes [19]. CircPABPN1 can bind to HuR in order to suppress translation of PABPN1 mRNA [35]. CircANRIL competitively recruit PES1 for inhibition of ribosome biogenesis [36]. CircFoxo3 interact with various RBPs and participate in cardiomyocyte ageing processes and progression of cell cycle [37]. CircAmotl1 can protect cardiomyocytes and promote cell proliferation and also wound healing through binding to: PDK1, AKT1 and STAT3 [38,39].

## 3. CircRNAs in the Development of Heart Failure

Coronary artery occlusion leads to myocardial infarction (MI), which causes necrosis of a myocardial area, pathological remodelling (cardiac hypertrophy, cell death and fibrosis) and heart dysfunction [40,41]. Under such circumstances, myocardial contractility decreases and/or haemodynamic workload increases. The heart produces then a number of adaptive changes in order to maintain cardiac output. Cardiomyocyte hypertrophy and fibrosis are typical features of remodelling in the process of heart failure development [42,43]. A gradual loss of cardiomyocytes is the main cause of reduced heart function [44]. Apart from cell necrosis and apoptosis, autophagy also plays an important role in cardiomyocyte loss [45]. Furthermore, diabetes mellitus is also an important risk factor for the development of heart failure.

### 3.1. Myocardial Infarction

Recent evidence suggests that circRNAs play a role in the pathogenesis of acute myocardial infarction. CircRNA, MICRA (myocardial infarction-related circular RNA) plays a role in heart failure in patients with MI [46]. Vausort et al. demonstrated reduced MICRA levels in patients with MI. They also found that a lower MICRA level was related with a higher risk of left ventricular dysfunction [47]. It has been recently demonstrated that circular RNA originating from the related gene *Fndc3b* is downregulated in murine heart after MI [48]. Besides that, the presence of circFndc3b in cardiac endothelial cells enhanced the function of the endothelial cells and protected cardiomyocytes against death [48]. Cai et al. [49] demonstrated a cardioprotective role of circRNA Ttc3 via miR-15b sponging in myocardial infarction model in rodents.

CircHipk3 plays a significant role in many physiological and pathological conditions. The experiments conducted by Si et al. [50] provided evidence of an important role of circHipk3 in the regeneration of the heart after AMI. The expression of circHipk3 has also been found to be increased in the hearts of newborn mice. An important observation is that inhibition of circHipk3 expression also leads to inhibition of proliferation of cardiomyocytes. On the other hand, overexpression of this circRNA is associated with a decrease in cardiac dysfunction, which translates into a reduction in the area of fibrosis after AMI. This effect of CircHipk3 on cardiomyocyte proliferation is associated with increased acetylation of the Notch1 intracellular domain (N1ICD) leading to its stability [51]. CircHipk3 also acts as a sponge for miR-133a. In this conditions, activation of expression of connective tissue growth factor (CTGF) occurs and leads to the activation of endothelial cells [50]. Circular RNA-7 (ciRS-7) features the classic miRNA sponging action. It has been confirmed that ciRS-7 has over 70 miR-7 binding sites [25,51,52]. Geng et al. [53] reported increased ciRS-7 expression after myocardial infarction in the cardiac tissue. In addition, when ciRS-7 level was increased by a lentiviral-based overexpression in a rodent MI model an increase the extent of myocardial infarction was also observed. The authors have also stated in this paper that ciRS-7 by sponged miR-7 affect the axis of PARP/SP1, and thus regulates the apoptotic pathways [53].

### 3.2. Diabetic Cardiomyopathy

Diabetic heart disease or cardiomyopathy is defined as the presence of abnormal structure and function of the myocardium in diabetic individuals who do not have CAD, hypertension, or heart valve dysfunction [54]. Rubler et al. [55] reported this pathological condition for the first time in 1972. Initially, it is asymptomatic, but during this period, many changes occur at both the molecular and cellular levels, which result in diastolic dysfunction and then systolic dysfunction, leading to the development of heart failure [56,57].

After all, many clinical trials of cardiovascular disease in diabetic patients have already been carried out, effective pharmacological interventions in DCM remain inadequate. Both hyperglycemia and metabolic disorders lead to the overproduction of ROS, and then the accumulation of damaged DNA, proteins and lipids. These changes stimulate cellular dysfunction or apoptosis, causing chronic inflammation, fibrosis and vascular dysfunction, ultimately leading to DCM [58]. Inflammation, as a major feature of DCM pathogenesis, is generally significantly severe in both patients and animal models of type 1 and type 2 diabetes (T1 DM and T2 DM). At the same time, it was noted that in the diabetic heart there were significantly more inflammatory cells and pro-inflammatory factors [54,59,60]. In addition, vascular endothelial dysfunction is highlighted as a key and frequent cause of DCM.

Recent evidence indicates that circRNAs play an important role in the pathogenesis of DCM. An excellent review by Wan et al. [61] has recently been published, in which the authors described in detail the involvement of circRNA in diabetic cardiomyopathy. However, we present only the participation of some circRNA molecules in the pathogenesis of diabetic cardiomyopathy. Previous studies have indicated that miR-384 is involved in DM-related vascular disease [62]. An extremely interesting report was the observation that the RNA-binding protein, known as the Lin-28 homologue B (LIN28B), considered an oncogene, is strongly involved in diabetic complications [63,64]. Moreover, CircBPTF (circ_0045462)—a new circRNA, is back-coupled by the bromodomain finger transcription factor PHD (BPTF) and circBPTF expression is strongly elevated in human umbilical vein endothelial cells (HUVEC) treated with high glucose concentrations [65]. Disorders such as oxidative stress, inflammation or endothelial dysfunction underlie dysregulation of circBPTF, miR-384 and LIN28B [61,66]. Bioinformatics analysis showed that miR-384 is a target for circBPTF and is decreased in HUVEC subjected to high glucose concentrations. The experimental studies carried out for this purpose clearly confirmed that LIN28B is a target for miR-384 and at the same time miR-384 can positively regulate the expression of LIN28B. In the high glucose (HG)-induced HUVEC model, attention was paid to increased cell viability and inhibition of cell apoptosis, release of pro-inflammatory cytokines and oxidative stress. At this experimental conditions, expression of miR-384 and LIN28B was regulated by lowering circBPTF [61,66]. In addition, it was found that the action of the miR-384/LIN28B axis in HUVEC by lowering circBPTF was able to attenuate the inflammation induced by high glucose levels and oxidative stress. Not surprisingly, oxidative stress, inflammation and endothelial dysfunction play an important role in DCM, while the circular RNA: circBPTF indicates the possibility of an effective intervention (Table 1).

### 3.3. Cardiac Hypertrophy

Cardiomyocyte hypertrophy is one of the basic adaptive mechanisms after heart injuries due to myocardial infarction [70]. Heart-related CircRNA (HRCR) has been known for inhibition of cardiac hypertrophy through miRNA-223 sponging [55]. Wang et al. [71] observed in transgenic mice that miRNA-223 overexpression caused cardiac hypertrophy, while in mice with miRNA-223 knock-out no signs of cardiac hypertrophy were found. HRCR overexpression abolished cardiac hypertrophy in miR-223 transgenic murine model. These results suggest that HRCR acts as a miR-223 sponge in order to reduce the hypertrophic response of the heart. In another paper, Li et al. [72] reported that circRNA_000203 induced cardiac hypertrophy through inhibition of miR-26b-5p and miR-140-3p. Moreover, a significantly increased cardiac hypertrophy and cardiac function impairment were found in a model of Ang-II-induced hypertrophy in transgenic circRNA_000203 (Tg-circ203) mice. CircRNA_000203 acts as a sponge for miR-26b-5p and as a sponge for miR-140-3p and increases the level of GATA4-binding protein, which leads to increased cardiac hypertrophy [72].

Lim et al. [73] studied the circSIc8a1 circular RNA, present in high amounts in the cardiomyocytes, and its role in cardiac hypertrophy. The authors, using a murine cardiac hypertrophy model with pressure overload, knocking down circSIc8a1, observed a suppressed cardiac hypertrophy. On the other hand, an AAV9-mediated application forced a circSIc8a1 expression increase, causing myocardial hypertrophy followed by myocardial failure. In such situations an in vivo application of artificially generated circular RNA sponges supplied by adenovirus-mediated way to the heart as competitive inhibitors of miR-132 and miR-212, proved helpful, resulting in restoration of the cardiac function and preventing cardiac hypertrophy as a consequence of trans-aortic constriction (TAC) injury [74].

### 3.4. Cardiac Fibrosis

Cardiac fibrosis is another form of heart remodelling and is frequently characterised by proliferation and activation of myofibroblasts followed by excessive deposition of extracellular matrix (ECM) components in the myocardium, leading to heart failure development [75,76]. Macrophages provide complete homeostasis in the myocardium, but when a cardiac injury occurs, macrophages quickly proliferate and serve as suppliers of inflammation and fibrosis mediators through release of various inflammatory cytokines or many growth factors, and through deposition of ECM components [77,78]. In cardiac fibrosis, circNFIB interacts with endogenous miR-433 to counteract proliferation of fibroblasts and stops the intensification of cardiac fibrosis [79]. Conversely, circHIPK3 promotes fibroblast accumulation through suppression of miR-29b-3p [80]. In cardiac fibroblast, circRNA affects the release pro-inflammatory cytokines. It has been shown, circ_010567 acts as the miR-141 sponge. In consequence the interaction of miR-141 with TGF-β1 (tumor growth factor β1), changes the expression of profibrotic proteins [68,81]. Increased expression of circ_000203 in diabetic and Ang II-induced murine fibroblast was observed. This circRNA is able to interacts with miR-26b-5p and leading to inhibition expression of CTGF and α-SMA (alpha smooth muscle actin) in cardiac fibrosis. Thus, as a result of miR-26b-5p inhibition, extensive fibrosis occurs [67,81].

### 3.5. Autophagy and Apoptosis

Autophagy is an evolution-preserved important process of intracellular material turnover. In the process some damaged proteins or organelles are wrapped in a double membrane structure of autophagous vesicles and then absorbed into lysosomes or vacuoles for degradation and recycling [82,83]. The body of evidence is growing that autophagy is associated with heart failure [84]. An analysis of circRNAs micromatrix with the use of murine circRNA_006636 as an internal reference gene, demonstrated a significantly reduced expression of autophagy-related circRNA (ACR) in murine heart with ischaemic-reperfusion injury. Besides that, exogenous ACR can also improve myocardial dysfunction caused by ischaemic/reperfusion injury. Further studies have demonstrated that ACR can activate Pink1 expression through binding to Dnmt3B and blocking of methylation of the DNA of Pink1 promoter by Dnm3B. Later, an activation of FAM65B expression and inhibition of cardiomyocyte autophagy occur in the process of heart failure development [85].

Apoptosis is a mechanism of spontaneous and arranged cell death, controlled by genes in order to maintain a stable internal environment [86,87]. Wencker et al. [87] discovered in a murine model that inhibition of cardiomyocyte apoptosis prevented to a significant extent the development of heart dilation and systolic dysfunction, what suggests that apoptosis of the cardiomyocytes may be the causal mechanism of heart failure.

In recent years, it has been demonstrated that circRNAs regulate heart failure, participating in the process of cardiomyocyte apoptosis. In healthy individuals, the heart is provided with mitochondrial machinery supplying it with adequate amount of energy, fully meeting the requirements of cardiomyocyte functioning. In the case of dysfunction concerning mitochondrial fission as a mechanism of mitochondrial quality control, it can lead to the development of various heart diseases including myocardial infarction or heart failure [88,89]. MTP18 is a nuclear-encoded mitochondrial membrane protein, controlling mitochondrial fission [90]. Wang et al. [88] have demonstrated that MTP18 mediates mitochondrial fission and apoptosis of the cardiomyocytes. They have discovered that miR-652-3p regulates MTP18 expression at translation level but does not change MTP18 mRNA expression. Further studies have revealed that circRNA, mm9-circ-016597 (MFACR), which is significantly stronger expressed in murine heart after ischaemia/reperfusion, can act as a miR-652-3p sponge and regulate the expression and activity of MTP18. In this way a regulatory pathway was obtained of mitochondrial fission and apoptosis in the cardiomyocytes, consisting of MFACR/miR-652-3p/MTP18 [88] (Table 2).

## 4. Conclusions

Recently, many circRNAs have been identified, characteristic of patients with cardiovascular diseases, including particularly heart failure. CircRNAs in various cell types fulfil diverse functions both under physiological and pathological conditions, such as silencing of transcription and translation and/or suppression of definite mRNAs. Nevertheless, the still insufficient description of circRNAs molecules remains a growing problem in the field of circRNAs studies; frequently, even an incoherent nomenclature of circRNAs is encountered. Detailed information is also lacking on the genomic location of each circRNA, and the functionality of many circRNAs still remains a great question mark.

CircRNAs have a crucial potential as molecular markers for diagnosing, prognostication and monitoring of diseases, not only cardiovascular ones, but also tumours and autoimmune disorders. That results from the fact that circRNA molecules are degraded by exonucleases, in view of their closed circular structure. It has been found that circRNAs are present in significant amounts in many body fluids, including plasma [94], serum [95] and saliva [96], and also in exosomes [97]. In view of their so widespread occurrence, circRNA molecules can open in the future many new diagnostic and therapeutic possibilities. CircRNAs are increasingly recognized as a promising biomarker and may play a significant role in therapeutic targets in a variety of diseases [98,99]. Currently, circRNA has become the subject of modern research, especially in the field of effective diagnostics and therapy, thanks to the presence of unique structural features, biological properties and biological functions [100]. Additionally, given their structural stability and presence in exosomes, circRNAs can act in an autocrine, paracrine, and even endocrine fashion. CircRNAs are widely distributed not only in cells but also in the extracellular space and various body fluids. The above-mentioned characteristics of circRNA confirm that they can be ideal biomarkers. Each newly discovered type of RNA molecule was assessed for biomarker potential. A bioinformatic analysis and computational biology based on the concepts of systems biology allowed the discovery of messenger RNA [101], miRNA [5,6,102] and lncRNA [8,9,103,104] as biomarkers of cardiovascular disease. Blood transcripts have been shown to be a rich inventory of potential biomarkers of cardiovascular disease [105]. However, the role of circRNA biomarkers in cardiovascular disease has only recently been suggested. Salgado-Somoza et al. [47] examined patients after myocardial infarction and showed that circRNA appears to be a predictor of the development of heart failure within several months after the event. The circRNA MICRA also provided much prognostic information for other known biomarkers and risk factors [46,47].

## 5. Future Directions and Perspectives

The strategy of circRNA-based therapies depends on their stability, differential expression in different organs, and specificity for a particular disease [106]. As with miRNAs, the likelihood of producing significant and undesirable off-target effects can greatly reduce the use of circRNA as a therapeutic agent. In addition, it is worth noting that the greater the specificity of the circRNA for a given organ or disease, the less likely it is to have out-of-target effects. A prerequisite and at the same time very important in RNA-based targeted therapy for specific diseases is an understanding of the role of specific circRNAs. On the other hand, the use of ncRNA conjugation to tissue-specific antibodies and/or peptides increases tissue/cell specificity while decreasing off-target effects. However, more intensive research is needed to determine both the therapeutic potential and the therapeutic use of circular RNAs.

## Figures and Tables

**Table 1:** CircRNAs involvement in the pathogenesis of diabetic cardiomyopathy.

Diabetic Cardiomyopathy					
CircRNAs	Expression	Targets	Place of Action	Effect	References
CircBPTF	↑	miR-384 ↓	Endothelial cells	Endothelial dysfunction	[65,66]
CircRNA_000203	↑	miR-26b-5p ↓	Myocardial fibroblasts	Fibrosis	[67]
CircRNA_010567	↑	miR-141 ↓	Myocardial fibroblasts	Fibrosis	[68]
CACR	↑	miR-214-3p ↓	Cardiomiocytes	Inflammation Pyroptosis Apoptosis	[69]

**Table 2:** CircRNAs involved in the development of heart failure.

CircRNAs	Expression	Targets	Subcellular Localization	Affected Pathway	Effect	References
**Cardiac hypertrophy**						
Circ-HRCR	↓	miR-223	Cytoplasm	ARC axis	Reduces hypertrophy and heart failure	[70]
CircRNA_000203	↑	miR-140-3p or miR-26b-5p	Cytoplasm	GATA4 axis	Exacerbates cardiac hypertrophy Excludes the anti-fibrotic effect of miR-26b in mouse CFs	[72] [67]
CiRS-7	↑	miR-7	Cytoplasm	PARP/SP1	Worsens cardiac function	[53]
**Cardiac fibrosis**						
CircNF1B	↓	miR-433	Cytoplasm	Fibroblast proliferation	Supports protection against cardiac fibrosis, attenuates fibrosis of cardiac	[79]
CircHIPK3	↑	miR-29b-3p or miR-17-3p	Cytoplasm	ADCY6 axis	Advances fibrosis of cardiac by up-regulating fibrosis-associated genes	[80,91]
CircRNA_010567	↑	miR-141/TGF	Cytoplasm	β1 axis	Reduces fibrosis-associated Col I, Col III and α-SMA protein expression	[68]
CircRNA_005647	↓	miR-27b-3p	Cytoplasm	DAPK	Reduces expressions of fibrosis-related genes	[81]
Circ_LAS1L	↓	miR-125b	Cytoplasm	SFRP5 axis	Reduces the activation, proliferation and migration of cardiac fibroblasts	[92]
CircPAN3	↑	miR-221	Cytoplasm	FoxO3/TG7	Advances cardiac fibrosis post-MI	[93]
**Autophagy and apoptosis**						
CircACR	↓	Pink1	Cytoplasm	FAM65B axis	Extinguish autophagy to attenuate myocardial ischemia/reperfusion injury	[85]
Circ-MFACR	↑	miR-652-3p	Cytoplasm	MTP18 axis	Advances mitochondrial fission and cardiomyocyte apoptosis	[71]
Circ_Ttc3	↓	miR-15b	Cytoplasm	Arl2 axis	Causes cardioprotection by reducing cardiomyocyte apoptosis	[49]
CircFndc3b	↑	RNA binding protein	Nucleus	FUS/ VEGF-A axis	Extinguish cardiomyocyte apoptosis, improves neovascularization and pick up left ventricular function in post-MI events	[48]
CircNfix	↑	miR-214	Cytoplasm	YBX1/NEDD4L	Extinguish cardiomyocyte proliferation, angiogenesis and increases CM apoptosis	[22]

ADCY6—adenylate cyclase type 6; ARC—apoptosis repressor with CARD domain; Arl2—ADP ribosylation factor-like protein 2; ATG7—Autophagy related 7 protein; DAPK—death associated protein kinase; FoxO3—forkhead box O3; FUS—fused in sarcoma; Gata4—GATA binding protein 4; NEDD4L—neural precursor cell expressed developmentally downregulated gene 4-like; PARP—poly (ADP ribose) polymerase; Pink1—PTEN induced putative kinase 1; SFRP5—secreted frizzled-related protein 5; SP1—stimulatory protein 1;VEGF—vascular endothelial growth factor; YBX1—Y box binding protein 1.

## Data Availability

Not applicable.

## References

[1] ENCODE Project Consortium (2012). An integrated encyclopedia of DNA elements in the human genome. Nature.

[2] Barrett S.P., Salzman J. (2016). Circular RNAs: Analysis, expression and potential functions. Development.

[3] Chen X., Han P., Zhou T., Guo X., Song X., Li Y. (2016). circRNADb: A comprehensive database for human circular RNAs with protein-coding annotations. Sci. Rep..

[4] Townsend N., Wilson L., Bhatnagar P., Wickramasinghe K., Rayner M., Nichols M. (2016). Cardiovascular disease in Europe: Epidemiological update. Eur. Heart J..

[5] Beermann J., Piccoli M.T., Viereck J., Thum T. (2016). Non-coding RNAs in development and disease: Background, mechanisms, and therapeutic approaches. Physiol. Rev..

[6] Goretti E., Wagner D.R., Devaux Y. (2014). miRNAs as biomarkers of myocardial infarction: A step forward towards personalized medicine?. Trends Mol. Med..

[7] Sygitowicz G., Maciejak-Jastrzębska A., Sitkiewicz D. (2020). MicroRNAs in the development of left ventricular remodeling and postmyocardial infarction heart failure. Pol. Arch. Intern Med..

[8] Gomes C.P.C., Spencer H., Ford K.L., Michel L.Y.M., Baker A.H., Emanueli C., Balligand J.L., Devaux Y. (2017). Cardiolinc network. The function and therapeutic potential of long non-coding RNAs in cardiovascular development and disease. Mol. Ther. Nucleic Acids.

[9] Greco S., Somoza A.S., Devaux Y., Martelli F. (2018). Long noncoding RNAs and cardiac disease. Antioxid. Redox. Signal..

[10] Zhang X.O., Wang H.B., Zhang Y., Lu X., Chen L.L., Yang L. (2014). Complementary sequence-mediated exon circularization. Cell.

[11] Conn S.J., Pillman K.A., Toubia J., Conn V.M., Salmanidis M., Phillips C.A., Roslan S., Schreiber A.W., Gregory P.A., Goodall G.J. (2015). The RNA binding protein quaking regulates formation of circRNAs. Cell.

[12] Barrett S.P., Wang P.L., Salzman J. (2015). Circular RNA biogenesis can proceed through an exon-containing lariat precursor. eLife.

[13] Kelly S., Greenman C., Cook P.R. (2015). A Papantonis. Exon skipping is correlated with exon circularization. J. Mol. Biol..

[14] Monat C., Quiroga C., Laroche-Johnston F., Cousineau B. (2015). The Ll.LtrB intron from Lactococcus lactis excises as circles in vivo: Insights into the group II intron circularization pathway. RNA.

[15] Li Z., Huang C., Bao C., Chen L., Lin M., Wang X., Zhong G., Yu B., Hu W., Dai L. (2015). Exon-intron circular RNAs regulate transcription in the nucleus. Nat. Struct. Mol. Biol..

[16] Werfel S., Nothjunge S., Schwarzmayr T., Strom T.M., Meitinger T., Engelhardt S. (2016). Characterization of circular RNAs in human, mouse and rat hearts. J. Mol. Cell Cardiol..

[17] Lu Q., Liu T., Feng H., Yang R., Zhao X., Chen W., Jiang B., Qin H., Guo X., Liu M. (2019). Circular RNA circSLC8A1 acts as a sponge of miR-130b/miR-494 in suppressing bladder cancer progression via regulating PTEN. Mol. Cancer.

[18] Zhu Y.J., Zheng B., Luo G.J., Ma X.K., Lu X.Y., Lin X.M., Yang S., Zhao Q., Wu T., Li Z.X. (2019). Circular RNAs negatively regulate cancer stem cells by physically binding FMRP against CCAR1 complex in hepatocellular carcinoma. Theranostics.

[19] Ashwal-Fluss R., Meyer M., Pamudurti N.R., Ivanov A., Bartok O., Hanan M., Evantal N., Memczak S., Rajewsky N., Kadener S. (2014). CircRNA biogenesis competes with pre-mRNA splicing. Mol. Cell.

[20] Zhang M., Huang N., Yang X., Luo J., Yan S., Xiao F., Chen W., Gao X., Zhao K., Zhou H. (2018). A novel protein encoded by the circular form of the SHPRH gene suppresses glioma tumorigenesis. Oncogene.

[21] Hansen T.B., Jensen T.I., Clausen B.H., Bramsen J.B., Finsen B., Damgaard C.K., Kjems J. (2013). Natural RNA circles function as efficient microRNA sponges. Nature.

[22] Huang S., Li X., Zheng H., Si X., Li B., Wei G., Li C., Chen Y., Chen Y., Liao W. (2019). Loss of super-enhancer-regulated circRNA Nfix induces cardiac regeneration after myocardial infarction in adult mice. Circulation.

[23] Hall L.F., Climent M., Quintavalle M., Farina F.M., Schorn T., Zani S., Carullo P., Kunderfranco P., Civilini E., Condorelli G. (2019). Circ_Lrp6, a circular RNA enriched in vascular smooth muscle cells, acts as a sponge regulating miRNA-145 function. Circ. Res..

[24] Zhang L., Zhang Y., Wang Y., Zhao Y., Ding H., Li P. (2020). Circular RNAs: Functions and clinical significance in cardiovascular disease. Front. Cell Dev. Biol..

[25] Memczak S., Jens M., Elefsinioti A., Torti F., Krueger J., Rybak A., Maier L., Mackowiak S.D., Gregersen L.H., Munschauer M. (2013). Circular RNAs are a large class of animal RNAs with regulatory potency. Nature.

[26] Wong C.H., Lou U.K., Li Y., Chan S.L., Tong J.H., To K.F., Chen Y. (2020). CircFOXK2 promotes growth and metastasis of pancreatic ductal adenocarcinoma by complexing with RNA-binding proteins and sponging miR-942. Cancer Res..

[27] Dong K., He X., Su H., Fulton D.J.R., Zhou J. (2020). Genomic analysis of circular RNAs in heart. BMC Med. Genom..

[28] Pan X., Shen H.B. (2017). RNA-protein binding motifs mining with a new hybrid deep learning based cross-domain knowledge integration approach. BMC Bioinform..

[29] Pan X., Rijnbeek P., Yan J., Shen H.B. (2018). Prediction of RNA-protein sequence and structure binding preferences using deep convolutional and recurrent neural networks. BMC Gen..

[30] Pan X., Shen H.B. (2018). Predicting RNA-protein binding sites and motifs through combining local and global deep convolutional neural networks. Bioinformatics.

[31] Wang Z., Lei X., Wu F.X. (2019). Identifying cancer-specific circRNA-RBP binding sites based on deep learning. Molecules.

[32] Bhuyan R., Bagchi A. (2020). Prediction of the differentially expressed circRNAs to decipher their roles in the onset of human colorectal cancers. Gene.

[33] Wawrzyniak O., Zarebska Z., Kuczynski K., Gotz-Wieckowska A., Rolle K. (2020). Protein-related circular RNAs in human pathologies. Cells.

[34] Zang J., Lu D., Xu A. (2020). The interaction of circRNAs and RNA binding proteins: An important part of circRNA maintenance and function. J. Neurosci. Res..

[35] Abdelmohsen K., Panda A.C., Munk R., Grammatikakis I., Dudekula D.B., De S., Kim J., Noh J.H., Kim K.M., Martindole J.L. (2017). Identification of HuR target circular RNAs uncovers suppression of PABPN1 translation by CircPABPN1. RNA Biol..

[36] Holdt L.M., Stahringer A., Sass K., Pichler G., Kulak N.A., Wilfert W., Kohlmaier A., Herbst A., Northoff B.H., Nicolaon A. (2016). Circular non-coding RNA ANRIL modulates ribosomal RNA maturation and atherosclerosis in humans. Nat. Commun..

[37] Du W.W., Yang W., Chen Y., Wu Z.K., Foster F.S., Yang Z., Li X., Yang B.B. (2017). Foxo3 circular RNA promotes cardiac senescence by modulating multiple factors associated with stress and senescence responses. Eur. Heart J..

[38] Yang Z.G., Awan F.M., Du W.W., Zeng Y., Lyu J., Wu D., Gupta S., Yang W., Yang B.B. (2017). The circular RNA interacts with STAT3, increasing its nuclear translocation and wound repair by modulating Dnmt3a and miR-17 function. Mol. Ther..

[39] Zeng Y., Du W.W., Wu Y., Yang Z., Awan F.M., Li X., Yang W., Zhang C., Yang Q., Yee A. (2017). A circular RNA binds to and activates AKT phosphorylation and nuclear localization reducing apoptosis and enhancing cardiac repair. Theranostics.

[40] Hou J., Kang Y.J. (2012). Regression of pathological cardiac hypertrophy: Signaling pathways and therapeutic targets. Pharmacol. Ther..

[41] Ladage D., Tilemann L., Ishikawa K., Correll R.N., Kawase Y., Houser S.R., Molkentin J.D., Hajjar R.J. (2011). Inhibition of PKCalpha/beta with ruboxistaurin antagonizes heart failure in pigs after myocardial infarction injury. Circ. Res..

[42] Tham Y.K., Bernardo B.C., Ooi J.Y.Y., Weeks K.L., McMullen J.R. (2015). Pathophysiology of cardiac hypertrophy and heart failure: Signaling pathways and novel therapeutic targets. Arch. Toxicol..

[43] Ma Y., de Castro Bras L.E., Toba H., Iyer R.P., Hall M.E., Winniford M.D., Lange R.A., Tyagi S.C., Lindsay M.L. (2014). Myofibroblasts and the extracellular matrix network in postmyocardial infarction cardiac remodeling. Pflug. Arch.-Eur. J. Physiol..

[44] Takemura G., Kanamari H., Okada H., Miyazaki N., Watanabe T., Tsujimoto A., Goto K., Maruyama R., Fujiwara T., Fujiwara H. (2018). Anti-apoptosis in nonmyocytes and pro-autophagy in cardiomyocytes: Two strategies against postinfarction heart failure through regulation of cell death/degeneration. Heart Fail. Rev..

[45] Kroemer G., Galluzzi L., Vandenabeele P., Abrams J., Alnemri E.S., Baehrecke E.H., Blagosklonmy M.V., El-Deiry W.S., Golstein P., Green D.R. (2009). Classification of cell death: Recommendations of the Nomenclature Committee on Cell Death 2009. Cell Death Differ..

[46] Salgado-Somoza A., Zhang L., Vausort M., Devaux Y. (2017). The circular RNA MICRA for risk stratification after myocardial infarction. IJC Heart Vasculat..

[47] Vausort M., Salgado-Somoza A., Zhang L., Leszek P., Scholz M., Teren A., Burkhardt R., Thiery J., Wagner D.R., Devaux Y. (2016). Myocardial infarction-associated circular RNA predicting left ventricular dysfunction. J. Am. Coll. Cardiol..

[48] Garikipati V.N.S., Verma S.K., Cheng Z., Liang D., Truongcao M.M., Cimini M., Yue Y., Huang G., Wang C., Benedict C. (2019). Circular RNA CircFndc3b modulates cardiac repair after myocardial infarction via FUS/VEGF-A axis. Nat. Commun..

[49] Cai L., Qi B., Wu X., Peng S., Zhou G., Wei Y., Xu J., Chen S., Liu S. (2019). Circular RNA Ttc3 regulates cardiac function after myocardial infarction by sponging miR-15b. J. Mol. Cell Cardiol..

[50] Si X., Zheng H., Wei G., Li M., Li W., Wang H., Guo H., Sun J., Li C., Zhong S. (2020). circRNA Hipk3 induces cardiac regeneration after myocardial infarction in mice by binding to Notch1 and miR-133a. Mol. Ther. Nucleic Acids.

[51] Rai A.K., Lee B., Hebbard C., Uchida S., Garikipati V.N.S. (2021). Decoding the complexicity of circular RNAs in cardiovascular disease. Pharmacol. Res..

[52] Chen L.L. (2020). The expanding regulatory mechanisms and cellular functions of circular RNAs. Nat. Rev. Mol. Cell Biol..

[53] Geng H.H., Li R., Su Y.M., Xiao J., Pan M., Cai X.X., Ji X.P. (2016). The circular RNA Cdr1as promotes myocardial infarction by mediating the regulation of miR-7a on its target genes expression. PLoS ONE.

[54] Jia G., Hill M.A., Sowers J.R. (2018). Diabetic Cardiomyopathy: An update of mechanisms contributing to this clinical entity. Circ. Res..

[55] Rubler S., Dlugash J., Yuceoglu Y.Z., Kumral T., Branwood A.W., Grishman A. (1972). New type of cardiomyopathy associated with diabetic glomerulosclerosis. Am. J. Cardiol..

[56] Chavali V., Tyagi S.C., Mishra P.K. (2013). Predictors and prevention of diabetic cardiomyopathy. Diabetes Metab. Syndr. Obes..

[57] Falcao-Pires I., Leite-Moreira A.F. (2012). Diabetic cardiomyopathy: Understanding the molecular and cellular basis to progress in diagnosis and treatment. Heart Fail Rev..

[58] Kayama Y., Raaz U., Jagger A., Adam M., Schellinger I., Sakamoto M., Suzuki H., Toyama K., Spin J., Tsao P. (2015). Diabetic cardiovascular disease induced by oxidative stress. Int. J. Mol. Sci..

[59] Hu X., Bai T., Xu Z., Liu Q., Zheng Y., Cai L. (2017). Pathophysiological fundamentals of diabetic cardiomyopathy. Compr. Physiol..

[60] Varga Z.V., Giricz A., Liaudet L., Hask´o G., Ferdinandy P., Pacher P. (2015). Interplay of oxidative, nitrosative/nitrative stress, inflammation, cell death and autophagy in diabetic cardiomyopathy. Biochim. Biophys. Acta Mol. Cell Biol. Lipids.

[61] Wan H., Zhao S., Zeng Q., Tan Y., Zhang C., Liu L., Qu S. (2021). CircRNAs in diabetic cardiomyopathy. Clin. Chim. Acta.

[62] Xia F., Sun J.J., Jiang Y.Q., Li C.F. (2019). MicroRNA-384-3p inhibits retinal neovascularization through targeting hexokinase 2 in mice with diabetic retinopathy. J. Cell Physiol..

[63] Ma L., Zhao Q., Chen W., Zhang Y. (2018). Oncogene Lin28B increases chemosensitivity of colon cancer cells in a let-7-independent manner. Oncol. Lett..

[64] Brennan E., Wang B., McClelland A., Mohan M., Marai M., Beuscart O., Derouiche S., Gray S., Pickering R., Tikellis C. (2017). Protective effect of let-7 miRNA family in regulating inflammation in diabetes-associated atherosclerosis. Diabetes.

[65] Jin G., Wang Q., Hu X., Li X., Pei X., Xu E., Li M. (2019). Profiling and functional analysis of differentially expressed circular RNAs in high glucose-induced human umbilical vein endothelial cells. FEBS Open Biol..

[66] Zhang W., Sui Y. (2020). CircBPTF knockdown ameliorates high glucose-induced inflammatory injuries and oxidative stress by targeting the miR-384/LIN28B axis in human umbilical vein endothelial cells. Mol. Cell Biochem..

[67] Tang C.M., Zhang M., Huang L., Hu Z.Q., Zhu J.N., Xiao Z., Zhang Z., Lin Q.X., Zheng X.L., Yang M. (2017). CircRNA_000203 enhances the expression of fibrosis-associated genes by derepressing targets of miR-26b-5p, Col1a2 and CTGF, in cardiac fibroblasts. Sci. Rep..

[68] Zhou B., Yu J.W. (2017). A novel identified circular RNA, circRNA_010567, promotes myocardial fibrosis via suppressing miR-141 by targeting TGF-β1. Biochem. Biophys. Res. Commun..

[69] Yang F., Li A., Qin Y., Che H., Wang Y., Lv J., Li Y., Li H., Yue E., Ding X. (2019). A novel circular RNA mediates pyroptosis of diabetic cardiomyopathy by functioning as a competing endogenous RNA. Mol. Ther. Nucleic Acids..

[70] Nakamura M., Sadoshima J. (2018). Mechanisms of physiological and pathological cardiac hypertrophy. Nat. Rev. Cardiol..

[71] Wang K., Long B., Liu F., Wang J.X., Liu C.Y., Zhao B., Zhou L.Y., Sun T., Wang M., Yu T. (2016). A circular RNA protects the heart from pathological hypertrophy and heart failure by targeting miR-223. Eur. Heart J..

[72] Li H., Xu J.D., Fang X.H., Zhu J.N., Yang J., Pan R., Yuan S.J., Zeng N., Yang Z.Z., Yang H. (2020). Circular RNA circRNA_000203 aggravates cardiac hypertrophy via suppressing miR-26b-5p and miR-140-3p binding to Gata4. Cardiovasc. Res..

[73] Lim T.B., Aliwarga E., Luu T.D.A., Li Y.P., Ng S.L., Annadoray L., Sian S., Ackers-Johnson M.A., Foo R.S.Y. (2019). Targeting the highly abundant circular RNA circSlc8a1 in cardiomyocytes attenuates pressure overload induced hypertrophy. Cardiovasc. Res..

[74] Lavenniah A., Luu T.D.A., Li Y.P., Lim T.B., Jiang J., Ackers-Johnson M., Foo R.S.Y. (2020). Engineered circular RNA sponges act as miRNA inhibitors to attenuate pressure overload induced cardiac hypertrophy. Mol. Ther..

[75] Kong P., Christia P., Frangogiannis N.G. (2014). The pathogenesis of cardiac fibrosis. Cell Mol. Life Sci..

[76] Yong K.W., Li Y., Huang G., Lu T.J., WKZWSafwani Pingguan-Murphy B., Xu F. (2015). Mechanoregulation of cardiac myofibroblast differentiation: Implications for cardiac fibrosis and therapy. Am. J. Physiol. Heart Circ. Physiol..

[77] Kuwahara F., Kai H., Tokuda K., Takeya M., Takeshita A., Egashira K., Imaizumi T. (2004). Hypertensive myocardial fibrosis and diastolic dysfunction: Another model of inflammation?. Hypertension.

[78] Hulsmans M., Sam F., Nahrendorf M. (2016). Monocyte and macrophage contributions to cardiac remodeling. J. Mol. Cell Cardiol..

[79] Zhu Y., Pan W., Yang T., Meng X., Jiang Z., Tao L., Wang L. (2019). Upregulation of circular RNA CircNFIB attenuates cardiac fibrosis by sponging miR-433. Front Genet..

[80] Ni H., Li W.F., Zhuge Y., Xu S., Wang Y., Chen Y., Shen G., Wang F. (2019). Inhibition of circHIPK3 prevents angiotensin II-induced cardiac fibrosis by sponging miR-29b-3p. Int. J. Cardiol..

[81] Xu M., Xie F., Tang X., Wang T., Wang S. (2020). Insights into the role of circular RNA in macrophage activation and fibrosis disease. Pharmacol. Res..

[82] Mizushima N. (2007). Autophagy: Process and function. Genes Dev..

[83] Galluzzi L., Bravo-San Pedro J.M., Demaria S., Farmenti S.C., Kroemer G. (2017). Activating autophagy to potentiate immunogenic chemotherapy and radiation therapy. Nat. Rev. Clin. Oncol..

[84] Maejima Y., Kyoi S., Zhai P., Liu T., Li H., Ivessa A., Sciaretta S., Del Re D.P., Zabłocki D.K., Hsu C.P. (2013). Mst1 inhibits autophagy by promoting the interaction between Beclin1 and Bcl-2. Nat. Med..

[85] Zhou L.Y., Zhai M., Huang Y., Xu S., An T., Wang Y.H., Zhang R.C., Liu C.Y., Dong Y.H., Wang M. (2019). The circular RNA ACR attenuates myocardial ischemia/reperfusion injury by suppressing autophagy via modulation of the Pink1/FAM65B pathway. Cell Death Differ..

[86] Savitskaya M.A., Onishchenko G.E. (2015). Mechanisms of apoptosis. Biochemistry.

[87] Wencker D., Chandra M., Nguyen K., Miao W., Garantziotis S., Factor S.M., Shirani J., Amstrong R.C., Kitsis R.N. (2003). A mechanistic role for cardiac myocyte apoptosis in heart failure. J. Clin. Investig..

[88] Wang K., Gan T.Y., Li N., Liu C.Y., Zhou L.Y., Gao J.N., Chen C., Yan K.W., Ponnusamy M., Zhang Y.H. (2017). Circular RNA mediates cardiomyocyte death via miRNA dependent upregulation of MTP18 expression. Cell Death Differ..

[89] Sygitowicz G., Sitkiewicz D. (2022). Mitochondrial quality control: The role in cardiac injury. Front Biosci..

[90] Tondera D., Czauderna F., Paulick K., Schwarzer R., Kaufmann J., Santel A. (2005). The mitochondrial protein MTP18 contributes to mitochondrial fission in mammalian cells. J. Cell Sci..

[91] Deng Y., Wang J., Xie G., Zeng X., Li H. (2019). Circ-HIPK3 strengthens the effects of adrenaline in heart failure by miR-17-3p-ADCY6 axis. Int. J. Biol. Sci..

[92] Sun L.Y., Zhao J.C., Ge X.M., Zhang H., Wang C.M., Bie Z.D. (2020). Circ_LAS1L regulates cardiac fibroblast activation, growth, and migration through miR-125b/SFRP5pathway. Cell Biochem. Funct..

[93] Li F., Long T.Y., Bi S.S., Sheikh S.A., Zhang C.L. (2020). circPAN3 exerts a profibrotic role via sponging miR-221 through FoxO3/ATG7-activated autophagy in a rat model of myocardial infarction. Life Sci..

[94] Ouyang Q., Huang Q., Jiang Z., Zhao J., Shi G.P., Yang M. (2018). Using plasma circRNA_002453 as a novel biomarker in the diagnosis of lupus nephritis. Mol. Immunol..

[95] Wu J., Zhou Q., Niu Y., Chen J., Zhu Y., Ye S., Xi Y., Wang F., Qiu H., Bu S. (2019). Aberrant expression of serum circANRIL and hsa_circ_0123996 in children with Kawasaki disease. J. Clin. Lab. Anal..

[96] Bahn J.H., Zhang Q., Li F., Chan T.M., Lin X., Kim Y., Wong D.T., Xiao X. (2015). The landscape of microRNA, piwi-interacting RNA, and circular RNA in human saliva. Clin. Chem..

[97] Wang Y., Zhao R., Liu W., Wang Z., Rong J., Long X., Liu Z., Ge J., Shi B. (2019). Exosomal circHIPK3 released from hypoxia-pretreated cardiomyocytes regulates oxidative damage in cardiac microvascular endothelial cells via the miR-29a/IGF-1 pathway. Oxid. Med. Cell Longev..

[98] Tuo B., Chen Z., Dong Q., Chen C., Zhang H., Hu S., Sun Z. (2022). Role of exosomal circRNAs in tumor immunity and cancer progression. Cell Death Dis..

[99] Lu P., Ding F., Xiang Y.K., Hao L., Zhao M. (2022). Noncoding RNAs in cardiac hypertrophy and heart failure. Cells.

[100] Zhu L., Li N., Sun L., Zheng D., Shao G. (2021). Non-coding RNAs: The key detectors and regulators in cardiovascular disease. Genomics.

[101] Azuaje F.J., Dewey F.E., Brutsaert D.L., Devaux Y., Ashley E.A., Wagner D.R. (2012). Systems-based approaches to cardiovascular biomarker discovery. Circ. Cardiovasc. Genet..

[102] Devaux Y., Vausort M., McCann G.P., Zangrando J., Kelly D., Razvi N., Zhang L., Ng L.L., Wagner D.R., Squire L.B. (2013). Micro-RNA-150: A novel marker of left ventricular remodeling after acute myocardial infarction. Circ. Cardiovasc. Genet..

[103] Devaux Y., Zangrando J., Schroen B., Creemers E.E., Pedrazzini T., Chang C.P., Dorn G.W., Thum T., Heymans S. (2015). Long noncoding RNAs in cardiac development and ageing. Nat. Rev. Cardiol..

[104] Yuan T., Krishnan J. (2021). Non-coding RNAs in cardiac regeneration. Front. Physiol..

[105] Devaux Y., Creemers E.E., Boon R.A., Werfel S., Thum T., Engelhardt S., Dimmeler S., Squire I. (2017). Circular RNAs in heart failure. Eur. J. Heart Fail..

[106] Yang T., Long T., Du T., Chen Y., Dong Y., Huang Z.P. (2021). Circle the cardiac remodeling with circRNAs. Front. Cardiovasc. Med..

